# Expression and prognostic value of VEGFR-2, PDGFR-β, and c-Met in advanced hepatocellular carcinoma

**DOI:** 10.1186/1756-9966-32-16

**Published:** 2013-04-03

**Authors:** Jie Sheng Chu, Fei Jiao Ge, Bo Zhang, Yan Wang, Nicola Silvestris, Lie Jun Liu, Chuan Hua Zhao, Li Lin, Anna Elisabetta Brunetti, Ya Li Fu, Jun Wang, Angelo Paradiso, Jian Ming Xu

**Affiliations:** 1 The Affiliated Hospital Cancer Center, Academy of Military Medical Sciences, No.8 Dong Da Street. FengTai District, Beijing 100071, People’s Republic of China; 2 Department of Oncology, Jiujiang Cancer Hospital, Jiujiang 332000, Jiangxi, People’s Republic of China; 3 The Affiliated Hospital, Department of Pathology, Academy of Military Medical Science, No.8 Dong Da Street. FengTai District, Beijing 100071, People’s Republic of China; 4 Medical Oncology Unit – National Cancer Institute “Giovanni Paolo II”, Bari, Italy; 5 Scientific Direction - National Cancer Institute “Giovanni Paolo II”, Viale Orazio Flacco, 65, 70124, BARI, Italy

**Keywords:** Hepatocellular carcinoma, VEGFR-2, PDGFR-β, c-Met, Survival analysis, Sorafenib

## Abstract

**Background:**

We explore the clinical and prognostic significance of expression of vascular endothelial growth factor receptor (VEGFR)-2, platelet-derived growth factor receptor (PDGFR)-β, and c-Met in patients with hepatocellular carcinoma (HCC).

**Methods:**

The expression of VEGFR-2, PDGFR-β, and c-Met were determined by immunohistochemical examination of the tissues of 93 HCC patients. The relationships of these markers with clinicopathological factors and prognosis were then analyzed.

**Results:**

High expression of VEGFR-2, PDGFR-β, and c-Met was found in 86%, 19.4%, and 80.6% of patients, respectively. Expression of VEGFR-2 correlated with gender (P = 0.044), hepatitis B surface antigen positivity (P = 0.024), degree of tumor differentiation (P = 0.023), and hepatic cirrhosis (P = 0.026). Expression of PDGFR-β correlated with alpha-fetoprotein level (P = 0.029), tumor size (P = 0.033), and hepatic cirrhosis (P = 0.023). No significant correlations were identified between expression of c-Met and clinicopathological factors. Expression of PDGFR-β correlated with overall survival (P = 0.046) and expression of c-Met correlated with progression-free survival (P = 0.01).

**Conclusions:**

We found that in patients with HCC, high expression of VEGFR-2 correlates with chronic hepatitis B virus infection and hepatic cirrhosis. High expression of PDGFR-β is a predictor of poor prognosis. High expression of C-Met may predict therapeutic effectiveness of sorafenib in HCC patients.

## Background

Hepatocellular carcinoma (HCC) is the fifth most common malignant tumor worldwide, with over 600,000 new cases diagnosed each year, and is the third most common tumor-related cause of death [1]. Hepatitis B virus (HBV) infection, hepatitis C virus infection, and aflatoxin-induced oncogene activation and tumor suppressor gene inactivation are the main causes of HCC [2]. Surgical resection and liver transplantation may cure HCC, but about 85% of patients have locally advanced tumor or distant metastasis at the time of diagnosis, and are not suitable candidates for surgery [3]. Conventional chemotherapy for HCC has limited effectiveness, but recent breakthroughs in treatment with molecular-targeted drugs have been reported.

Abnormalities of intracellular signaling pathways which result in abnormal cell proliferation and apoptosis are one of the main mechanisms of HCC development. Many complex cellular signaling pathways are involved in tumor development and growth. These pathways include proteins such as vascular endothelial growth factor (VEGF), VEGF receptor (VEGFR), platelet-derived growth factor (PDGF), PDGF receptor (PDGFR), hepatocyte growth factor/c-Met, Ras/Raf/Mek/Erk, and PI3k/Ak/mTOR. High expression of VEGFR-2, PDGFR-β, and c-Met can be detected in many tumors, including HCC, but information regarding the relationships between expression of VEGFR-2, PDGFR-β, and c-Met and the clinicopathological factors and prognosis of HCC is very limited [4-7]. This study explored the relationships between expression of VEGFR-2, PDGFR-β, and c-Met and the clinicopathological factors and prognosis of HCC patients, aiming to provide reference information to assist with the diagnosis, evaluation of prognosis, and targeted therapy of HCC.

## Methods

Specimens were collected from 93 HCC patients treated at the Department of Digestive Oncology, Chinese People's Liberation Army 307 Hospital from January 2007 to October 2011. The specimens were collected from patients by biopsy and it was excluded if the biopsy specimen was too less. Sixty-five of these patients were taking sorafenib. All patients met the following inclusion criteria: (1) advanced stage HCC which was not suitable for surgery or local treatment, or had recurred after surgery or local treatment, (2) Child-Pugh class A or B, (3) Eastern Cooperative Oncology Group (ECOG) score 0 or 1, (4) at least one target lesion that had not been previously treated, (5) no local treatment for at least 4 weeks before baseline imaging, (6) availability of complete clinical and pathological data, including follow-up data. All specimens were fixed in 10% formaldehyde, embedded in paraffin, and cut into 4-μm thick slices before staining. Clinical and pathological data of all patients, and progression-free survival (PFS) and overall survival (OS) data of the 65 patients who took sorafenib, were obtained from medical records and telephone follow-ups, with a follow-up deadline of November 11, 2011. The clinical and pathological data collected included gender, age, hepatitis B surface antigen (HBsAg) status, serum alpha-fetoprotein (AFP) level, tumor number, tumor size, degree of tumor differentiation, Child-Pugh class, Barcelona Clinic Liver Cancer (BCLC) stage, presence of cirrhosis, ascites, tumor thrombus, and extrahepatic metastasis. The PFS and OS were defined as the time from initiation of sorafenib therapy to the time of disease progression detected by computed tomography or magnetic resonance imaging, or death, respectively.

### Immunohistochemical staining

Expression of VEGFR-2, PDGFR-β, and c-Met were determined by two-step PV-6000 immunohistochemistry staining. Specimen slices were dewaxed, rinsed in phosphate-buffered saline (PBS). Antigen retrieval was performed by placing the slides in a high pressure cooker in 0.01 mmol/L citrate buffer, pH 6.0, for 3 minutes at 100°C, followed by cooling for 20 min at room temperature, rinsing in PBS, treating with 3% hydrogen peroxide in deionized water for 10 min to block endogenous peroxidase, and rinsing again in PBS. Specimens were then incubated at 37°C for 1 hour with primary antibody against VEGFR-2 (dilution ratio 1:50; Santa Cruz Biotechnology Inc., Santa Cruz, CA), PDGFR-β (dilution ratio 1:40; Santa Cruz Biotechnology Inc., CA), and c-Met (rabbit anti-human c-Met monoclonal antibody working solution; Epitomics, California, US), followed by rinsing three times in PBS for 2 min each time. Specimens were incubated at 37°C for 20 min with universal IgG antibody-HRP polymer (Zhongshan Jinqiao Co., Beijing, China), and rinsed three times in PBS for 2 min each time. Specimens were placed in DAB solution for color development, rinsed with distilled water, stained again, dehydrated, and sealed with transparent strips. Primary antibodies were replaced with PBS to produce a negative control, and a known positive tissue slice was used as a positive control.

### Analysis of immunohistochemistry results

Two pathologists who were blind to diagnosis independently inspected the slices. The rate of agreement between the two pathologists was 95%. The scores from both pathologists were averaged to provide the final score for each case. A combination of positive cell count and staining intensity was used for scoring. Positive cell count was scored based on the average percentage of positive cells per 100 cells in 10 high-power fields, as follows: 0–10%, score 0; 11–25%, score 1; 26–50%, score 2; 51–75%, score 3; and >75%, score 4. Staining intensity was scored as follows: negative, score 0; faint yellow, score 1; yellow or deep yellow, score 2; brown or dark brown, score 3. The final score was obtained by multiplying the cell count and staining intensity scores. For VEGFR-2 and c-Met, a score of ≥ 5 was defined as high expression and a score of < 5 was defined low expression. For PDGFR-β, a score of ≥ 3 was defined as high expression group and a score of < 3 was defined as low expression.

### Statistical analysis

Statistical analyses were performed using SPSS software version 18.0. Categorical variables were compared using the χ^2^ test or Fisher's exact test. Survival rates were calculated using the Kaplan-Meier method. Univariate survival analyses were performed using the log-rank test, and multivariate survival analyses were performed using Cox’s proportional hazards model. P < 0.05 was considered statistically significant.

## Results

### VEGFR-2, PDGFR-β, c-MET

#### Expression of VEGFR-2, PDGFR-β, and c-MET in the tissues of HCC patients

**Figure 1:**
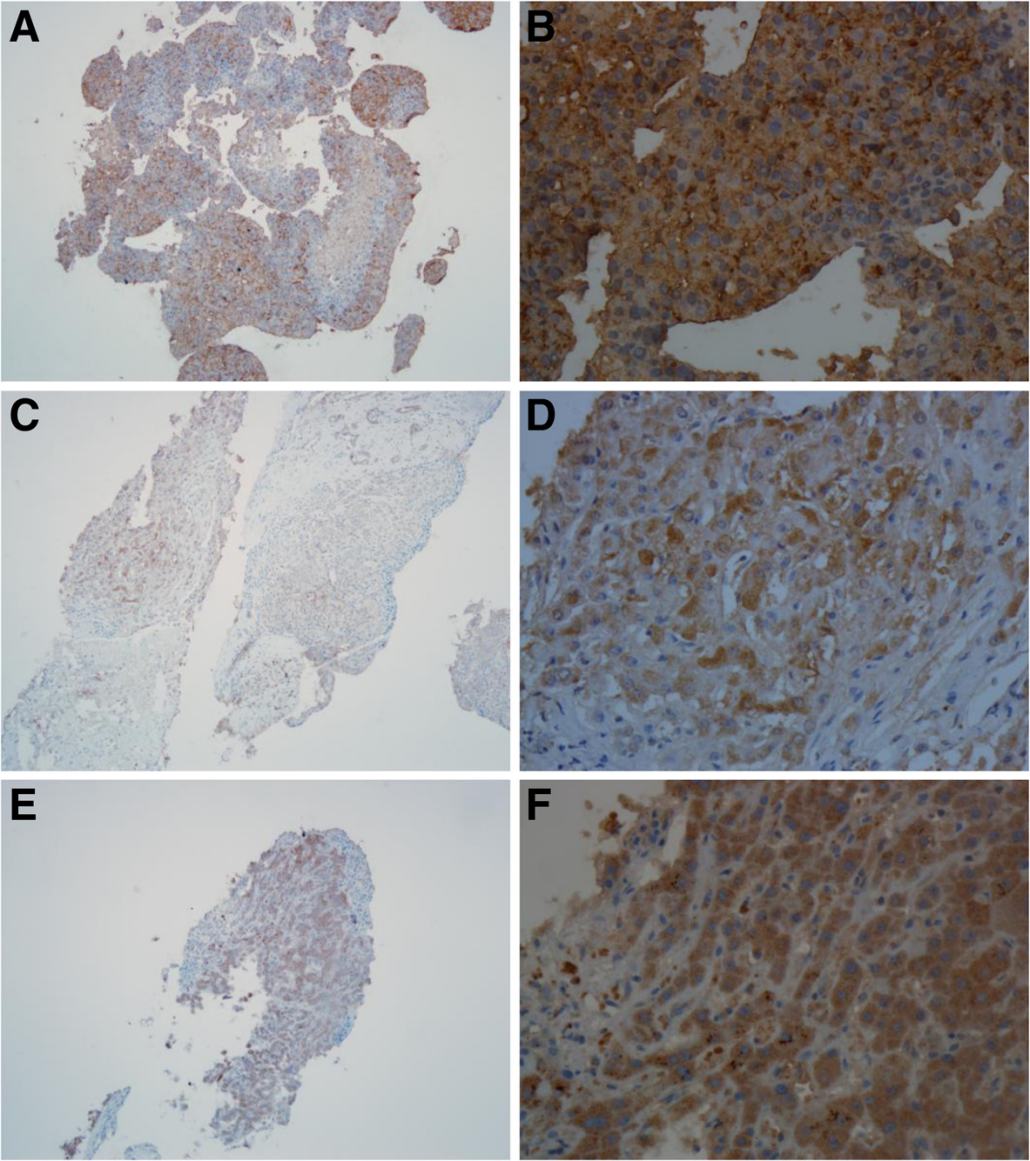
**Expression of VEGFR-2, PDGFR-β, and c-MET in hepatocellular carcinoma. A** Expression of cytoplasmic VEGFR-2 in hepatocellular carcinoma (PV-6000 staining, ×100). **B** Expression of VEGFR-2 (PV-6000 staining, ×400). **C** Expression of cytoplasmic PDGFR-β in hepatocellular carcinoma (PV-6000 staining, ×100). **D** Expression of PDGFR-β (PV-6000 staining, ×400). **E** Expression of cytoplasmic c-MET in hepatocellular carcinoma (PV-6000 staining, ×100). **F** Expression of c-MET (PV-6000 staining, × 400).

Expression of VEGFR-2, PDGFR- β, and c-MET was identified by immunohistochemical cytoplasmic staining with different colors varying from faint yellow to dark brown, with a granular or clustered distribution (Figure 1). High expression of VEGFR-2 was observed in 80 of 93 cases (86%), high expression of PDGFR- β was observed in 18 cases (19.4%), and high expression of c-Met was observed in 75 cases (80.6%).

### VEGFR-2, PDGFR-β, c-MET

#### Relationships between expression of VEGFR-2, PDGFR-β, and c-Met and clinicopathological factors

Expression of VEGFR-2 correlated with gender, HBsAg status, degree of tumor differentiation, and hepatic cirrhosis, but did not correlate with age, AFP level, tumor number, tumor size, Child-Pugh class, BCLC stage, ascites, tumor thrombus, or extrahepatic metastasis. High expression was more frequent in males than females (89.6% vs, 68.8%, P = 0.044), in HBsAg-positive patients than HBsAg-negative patients (89.9% vs. 64.3%, P = 0.024), in well-differentiated tumors than poorly-differentiated tumors (100% vs. 72.7%, P = 0.023), and in patients with cirrhosis than without cirrhosis (93.8% vs, 77.8%, P = 0.026). Expression of PDGFR-β correlated with AFP level, tumor number, and cirrhosis, but did not correlate with gender, age, HBsAg status, tumor size, degree of tumor differentiation, Child-Pugh class, BCLC stage, ascites, tumor thrombus, or extrahepatic metastasis. High expression of PDGFR-β was more frequent in patients with AFP > 400 IU/mL than with AFP ≤ 400 IU/mL (28.3% vs. 10.6%, P = 0.029), in patients with multiple tumors than with single tumors (25.0% vs. 6.9%, P = 0.033), and in patients without cirrhosis than with cirrhosis (28.9% vs. 10.4%, P = 0.023). There were no significant correlations between expression of c-MET and clinical or pathological factors, but high expression of c-Met tended to be more frequent in patients with BCLC stage C than stage B (84.9% vs 65%, P = 0.051) (Table 1).

**Table 1:** Relationships between expression of VEGFR-2, PDGFR-β, and C-met and clinicopathological factors

**Parameters**	**N**	**VEGFR-2**		**P**	**PDGFR-β**		**P**	**C-MET**		**P**
		**High**	**Low**		**High**	**Low**		**High**	**Low**	
N(%)	93	80(86.0)	13		18(19.4)	75		75(80.6)	18	
**Gender**										
Male	77	69(89.6)	8		15(19.5)	62		61(79.2)	16	
Female	16	11(68.8)	5	0.044	3(18.8)	13	0.627	14(87.5)	2	0.355
**Age**										
≤50	31	26(83.9)	5		6(19.4)	25		25(80.6)	6	
>50	62	54(87.1)	8	0.448	12(19.4)	50	0.602	50(80.6)	12	0.616
**HBsAg**										
Positive	79	71(89.9)	8		16(20.3)	63		63(79.7)	16	
Negative	14	9(64.3)	5	0.024	2(14.3)	12	0.461	12(85.7)	2	0.461
**AFP**(**IU**/**ML**)										
≤400	47	39(83.0)	8		5(10.6)	42		39(83.0)	8	
>400	46	41(89.1)	5	0.290	13(28.3)	33	0.029	36(78.3)	10	0.377
**Tumor number**										
Single	29	26(89.7)	3		2(6.9)	27		23(79.3)	6	
>1	64	54(84.4)	10	0.371	16(25.0)	48	0.033	52(81.3)	12	0.516
**Tumor size**(**cm**)										
≤5	16	13(81.3)	3		4(25.0)	12		13(81.3)	3	
>5	77	67(87.0)	10	0.394	14(18.2)	63	0.373	62(80.5)	15	0.627
Differentiation										
High	26	26(100)	0		7(26.9)	19		21(80.8)	5	
Middle	45	38(84.4)	7		6(13.3)	39		35(77.8)	10	
Low	22	16(72.7)	6	0.023	5(22.7)	17	0.340	19(86.4)	3	0.705
**Child**-**Pugh**										
A	82	70(85.4)	12		14(17.1)	68		64(78.0)	18	
B	11	10(90.9)	1	0.523	4(36.4)	7	0.134	11(100)	0	0.080
**BCLC**										
B	20	15(75.0)	5		2(10.0)	18		13(65.0)	7	
C	73	65(89.0)	8	0.111	16(21.9)	57	0.194	62(84.9)	11	0.051
**Hepatic cirrhosis**										
Yes	48	45(93.8)	3		5(10.4)	43		37(77.1)	11	
No	45	35(77.8)	10	0.026	13(28.9)	32	0.023	38(84.4)	7	0.263
Ascites										
Yes	19	17(89.5)	2		3(15.8)	16		17(89.5)	2	
No	74	63(85.1)	11	0.476	15(20.3)	59	0.470	58(78.4)	16	0.228
Tumor thrombus										
Yes	38	33(86.8)	5		10(26.3)	28		34(89.5)	4	
No	55	47(85.5)	8	0.551	8(14.5)	47(	0.126	41(74.5)	14	0.061
**Extrahepatic metastasis**										
Yes	48	43(89.6)	5		8(16.7)	40		40(83.3)	8	
No	45	37(82.2)	8	0.235	10(22.2)	35	0.339	35(77.8)	10	0.339

VEGFR-2, vascular endothelial growth factor receptor-2; PDGFR-β, platelet-derived growth factor receptor-β; C-MET, hepatocyte growth factor receptor; HbsAg, hepatitis B surface antigen; AFP, serum alpha-fetoprotein; BCLC, Barcelona Clinic Liver Cancer stage.

### HCC

#### Relationships between clinicopathological factors and prognosis

Univariate analyses showed that in the 65 patients who took sorafenib, PFS time correlated with age and OS time correlated with AFP level, tumor size, ascites, and tumor thrombus (Table 2). PFS time was longer in patients aged > 50 years than those aged ≤ 50 years (5.83 months vs. 4.00 months, P = 0.047). OS time was longer in patients with an AFP level ≤ 400 IU/mL than an AFP level > 400 IU/mL (11.13 months vs. 5.20 months, P = 0.022), in patients with a tumor size ≤ 5 cm than a tumor size > 5 cm (29.27 months vs. 5.87 months, P = 0.002), in patients without ascites than with ascites (8.97 months vs. 5.00 months, P = 0.049), and in patients without tumor thrombus than with tumor thrombus (11.37 months vs. 5.00 months, P = 0.005). Multivariate analyses showed that PFS time was independently correlated with age (P = 0.047) and OS time was independently correlated with HBsAg positivity (*P* = 0.037), AFP level (*P* = 0.015), and tumor size (*P* = 0.003).

**Table 2:** Univariate analyses of the relationships between clinicopathologic factors and survival

**Parameters**		**N**	**PFS**			**OS**		
			**Months**	***χ***^**2**^	**P**	**Months**	***χ***^**2**^	**P**
**Gender**	Male	55	4.433			7.400		
	Female	10	6.200	0.609	0.435	10.200	0.340	0.560
**Age**	≤50	22	4.000			5.867		
	>50	43	5.833	3.934	0.047	8.067	0.113	0.736
**HBsAg**	Positive	55	4.433			6.467		
	Negative	10	5.833	0.516	0.472	8.800	3.608	0.057
**AFP**(**IU**/**ml**)	≤400	31	7.000			11.133		
	>400	34	4.233	3.016	0.082	5.200	5.236	0.022
**Tumor number**	Single	18	5.600			8.967		
	>1	47	4.967	0.168	0.682	5.867	0.981	0.322
**Tumor size**(**cm**)	≤5	12	7.300			29.267		
	>5	53	4.367	3.792	0.051	5.867	9.834	0.002
Differentiation	High	17	6.200			5.233		
	Middle	33	4.367			8.967		
	Low	15	4.000	3.630	0.163	5.667	3.097	0.213
**Child**-**Pugh**	A	59	5.600			8.067		
	B	6	4.967	0.599	0.439	3.600	1.980	0.159
**BCLC**	B	7	5.633			10.500		
	C	58	4.433	3.527	0.060	7.400	0.274	0.600
**Hepatic cirrhosis**	Yes	34	4.967			6.533		
	No	31	4.433	0.002	0.965	8.967	0.194	0.659
Ascites	Yes	14	4.367			5.000		
	No	51	5.600	2.706	0.100	8.967	3.887	0.049
Tumor thrombus	Yes	28	3.000			5.000		
	No	37	5.833	2.800	0.094	11.367	8.067	0.005
**Extrahepatic metastasis**	Yes	41	4.367			6.467		
	No	24	5.600	0.878	0.349	8.967	0.017	0.897

PFS, progression-free survival; OS, overall survival; HbsAg, hepatitis B surface antigen; AFP, serum alpha-fetoprotein; BCLC, Barcelona Clinic Liver Cancer stage.

### VEGFR-2, PDGFR-β, c-MET

#### Relationships between expression of VEGFR-2, PDGFR-β, and c-MET and prognosis in patients who took sorafenib

**Figure 2:**
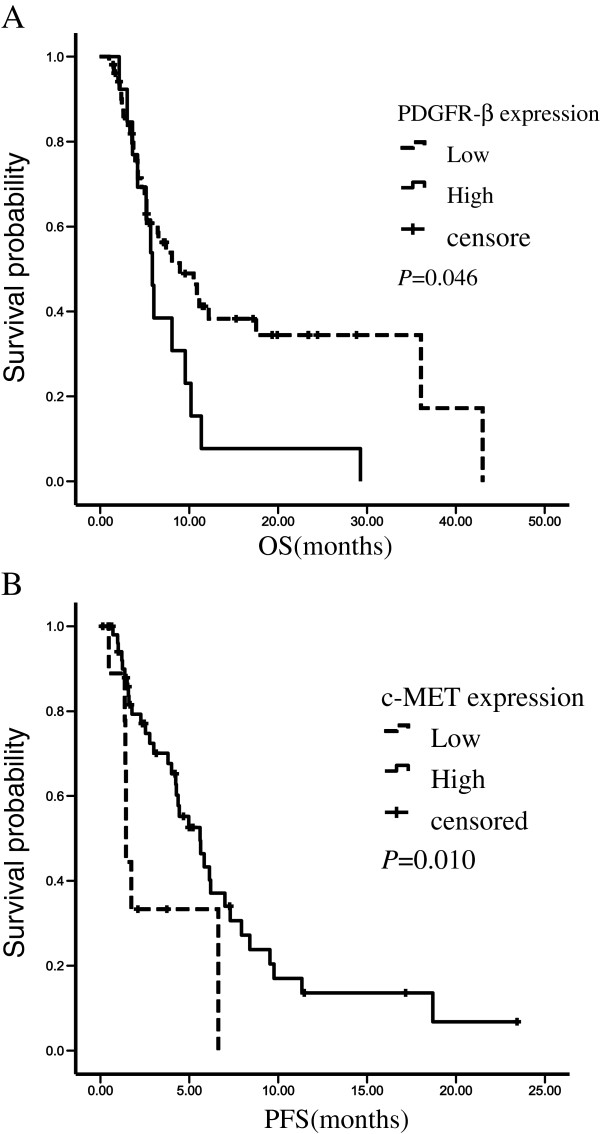
**Kaplan-Meier curves were plotted for PFS and OS.** Patients were divided into high-expression group and low-expression group by PDGFR-β expression or c-MET expression. **A**: Overall survival curves stratified by PDGFR-β expression (p=0.046). **B**: Progression-free survival curves stratified by c-MET expression (p=0.010). PFS, progression-free survival; OS, overall survival.

We used the Kaplan-Meier method and log-rank test to analyze the association between the expression of VEGFR-2, PDGFR-β, c-Met and prognosis. Among the 65 patients who took sorafenib, there was no significant difference between patients with high and low expression of VEGFR-2 in PFS time (*P* = 0.532) or OS time (*P* = 0.473). There was no significant difference between patients with high and low expression of PDGFR-β in PFS time (*P* = 0.246), but the median OS time was shorter in patients with high expression of PDGFR-β than low expression of PDGFR-β (5.87 months vs. 8.97 months, *P* = 0.046). The median PFS time was longer in patients with high expression of c-MET than low expression of c-MET (5.60 months vs. 1.43 months, *P* = 0.010), but there was no significant difference in OS time between patients with high and low expression of c-Met (Figure 2, Table 3).

**Table 3:** Relationships between expression of VEGFR-2,DGFR-β, and c-MET and prognosis in HCC patients who took sorafenib

	**N**	**PFS OS**					
		**Months**	***χ***^**2**^	**P**	**months**	***χ***^**2**^	**P**
**PDGFR**-**β**	65						
High	13	4.23			5.87		
Low	52	5.60	1.345	0.246	8.97	3.996	0.046
**VEGFR**-**2**	65						
High	58	4.97			7.40		
Low	7	7.93	0.391	0.532	11.37	0.514	0.473
**c**-**MET**	65						
High	55	5.60			8.97		
Low	10	1.43	6.558	0.010	6.47	0.930	0.335

VEGFR-2, vascular endothelial growth factor receptor-2; PDGFR-β, platelet-derived growth factor receptor-β; C-MET, hepatocyte growth factor receptor; PFS, progression-free survival; OS, overall survival.

## Discussion

The pathogenesis of HCC is believed to multifactorial. HBV infection and hepatic cirrhosis are known risk factors. In China, most patients with HCC have both HBV infection and cirrhosis. The specific signaling pathways and key proteins involved in the development of HCC have not been fully elucidated. Recently, a variety of proteins were confirmed to play an important role in the process, including VEGFR. Lian et al. [8] reported that hepatitis B x antigen was involved in the upregulation of VEGFR-3, which may be associated with the development of HCC. Corpechot et al. [9] reported that hepatocellular hypoxia led to angiogenesis and hepatic fibrosis in an animal model of cirrhosis, and that upregulation of the expression of VEGF and VEGFR-2 correlated with increased density of microvessels. Kornek et al. [10] reported that hepatic fibrosis may promote the development of HCC, and that VEGF-A and VEGFR-A may contribute to accelerated development of HCC. DeLeve et al. [11] reported that liver sinusoidal endothelial cells may secrete matrix metalloproteinase MMP2 and MMP9, and that MMP9 may cause the degradation of endothelial cells and thrombosis, resulting in sinusoidal obstruction syndrome. VEGF may promote MMP activity, thereby exacerbating the liver injury. Serum VEGF level is therefore related to the degree of liver injury. Ribero et al. [12] reported that patients with liver metastasis from colorectal cancer often had liver damage after taking oxaliplatin- or irinotecan-based chemotherapy, but the incidence and severity of this liver injury were significantly reduced when bevacizumab (VEGF McAb) was added. This indicates that high expression of VEGF in cirrhotic liver tissue is associated with the development and severity of cirrhosis. Inhibition of VEGF expression can reduce the incidence and severity of hepatic cirrhosis. This study also found high expression of VEGFR-2 in HCC patients with HBsAg positivity and hepatic cirrhosis. We speculate that expression of VEGF and VEGFR is upregulated in response to liver cell hypoxia resulting from HBV infection and cirrhosis, leading to angiogenesis. The resulting new blood vessels may provide the foundation for the development of tumor recurrence and metastasis. Prophylactic use of inhibitors of VEGF expression in patients with hepatic cirrhosis may prevent the development of cancer. This possibility requires further investigation.

HCC has a relatively poor prognosis, with a median survival time of only 6–9 months [1,13]. Although the Child-Pugh classification gives a relatively reliable indication of prognosis, some researchers prefer to use other indices such as the Cancer of the Liver Italian Program (CLIP) stage, BCLC stage, or Model for End-stage Liver Disease (MELD) stage. Although many studies have reported on the prognostic value of protein markers of liver cancer, there is no consensus regarding the use of these markers to predict prognosis. The results of the current study show that age, AFP level, tumor size, ascites, and tumor thrombus may correlate with the prognosis of HCC patients, and should probably be taken into account together with the Child-Pugh classification when considering prognosis. Our analyses found that OS time was shorter in patients with high expression of PDGFR-β than low expression of PDGFR-β, and that high expression of PDGFR-β correlated with AFP level > 400 IU/mL and multiple tumors. AFP level > 400 IU/mL and multiple tumors are indicators of poor prognosis in HCC patients, which suggests that high expression of PDGFR-β is also an indicator of poor prognosis. This conclusion is consistent with other recent research. Chen et al. [7] reported that simultaneous high expression of PDGFR-α, PDGFR-β, and VEGF was a predictor of poor prognosis in patients with HCC. Patel et al. [14] also reported that high expression of both PDGFR-α and PDGFR-β was an independent predictor of shorter OS time. Expression of PDGFR in patients with HCC may therefore be a useful indicator of prognosis.

Current comprehensive treatment of HCC includes molecular-targeted therapy. Sorafenib is currently the only molecular-targeted drug approved for the treatment of HCC. Two Phase III clinical trials [15,16] reported that sorafenib controlled disease in 43% and 35% of HCC patients, respectively, indicating that the majority of patients do not benefit from this treatment. As there are currently no known biological markers which can predict the efficacy of sorafenib treatment, evaluation of potential markers is very important. Researchers have evaluated many potential predictors of the effectiveness of sorafenib treatment, including clinical staging systems. Baek et al. [17] reported that the Cancer of the Liver Italian Program score or Okuda stage, together with performance status, could be used to predict the effectiveness of sorafenib treatment. Morimoto et al. [18] considered that the Glasgow Prognostic Score had a significant prognostic value. Song et al. [19] reported that ascites and distant metastasis predicted poor effectiveness, and that side effects of sorafenib treatment predicted good effectiveness. Pinter et al. [20] reported that low levels of AFP and ALT, Child-Pugh class B, and compensated cirrhosis were predictors of a good response to sorafenib treatment, and that AST level could be used to predict whether Child-Pugh class B patients would benefit from sorafenib treatment. Lee et al. [21] reported that patients with a low FDG uptake on positron-emission tomography might benefit from sorafenib treatment. Kondo et al. [22] reported that high expression of c-MET correlated with portal vein tumor thrombus, and that postoperative recurrence-free survival was significantly poorer in patients with high expression of c-Met than with low expression of c-Met. Expression of c-MET may be a predictor of postoperative recurrence in HCC patients. Our results did not show a significant difference in the frequency of portal vein tumor thrombus between patients with high and low expression of c-MET (89.5% vs. 74.5%, P = 0.061), which is probably because our assessment of tumor thrombus was based on imaging results, whereas Kondo et al. [22] based their assessment on pathological findings. Albig et al. [23] reported that high expression of c-Met may enhance the sensitivity of cancer tissues to hepatocyte growth factor, thereby increasing the invasiveness of cancer cells and the likelihood of metastasis. Combination of the results reported by Kondo et al. [22] and Albig et al. [23] suggests that patients with high expression of c-Met have a poor prognosis. However, our survival analyses show that in patients who took sorafenib, PFS time was longer in patients with high expression of c-Met than low expression of c-Met (5.60 months vs. 1.43 months, P = 0.010), suggesting that expression of c-MET may predict the effectiveness of sorafenib treatment in HCC patients. These results require further evaluation in studies with larger sample sizes and more carefully selected patients. From the statistic results, the median PFS time was longer in patients with high expression of c-MET than those in low expression of c-MET (5.60 months vs. 1.43 months, P = 0.010), but there was no significant difference in OS time between patients with high and low expression of c-Met, We considered the subsequent treatments after sorafenib may cause the discrepancy of longer PFS and no significant OS. In China, Patients with HCC usually received other treatments after failure to sorafenib, such as intervention therapy and Chinese herbal medicine and so on.

## Conclusions

In summary, our finding that HCC with hepatic cirrhosis is associated with high expression of VEGFR-2 provides new information to help our understanding of the development and treatment of hepatic cirrhosis. Age, AFP level, tumor size, ascites, and tumor thrombus may be useful prognostic indicators in HCC patients. High expression of PDGFR-β is an indicator of poor prognosis, and high expression of c-MET may predict therapeutic effectiveness of sorafenib treatment, allowing individualized treatment of HCC patients.

## Competing interests

The authors declare that they have no competing interests.

## Authors’ contributions

JSC, FJG, BZ, YW, LJL, CHZ, LL, YLF, JW and JMX designed the study, performed the experiments, and drafted the manuscript. AP,NS and AEB designed the study and supervised the experimental work. All authors read and approved the final manuscript.
